# Diagnosing pelvic osteomyelitis in patients with pressure ulcers: a systematic review comparing bone histology with alternative diagnostic modalities

**DOI:** 10.5194/jbji-6-21-2020

**Published:** 2020-08-26

**Authors:** Maria Chicco, Prashant Singh, Younatan Beitverda, Gillian Williams, Hassan Hirji, Guduru Gopal Rao

**Affiliations:** 1 Department of Microbiology, London North West University Healthcare NHS Trust, Northwick Park Hospital, Harrow, HA1 3UJ, UK; 2 Department of Trauma and Orthopaedics, Royal Free London NHS Foundation Trust, Barnet Hospital, Barnet, EN5 3DJ, UK; 3 Department of Geriatric Medicine, London North West University Healthcare NHS Trust, Northwick Park Hospital, Harrow, HA1 3UJ, UK; 4 Department of Cellular Pathology, London North West University Healthcare NHS Trust, Northwick Park Hospital, Harrow, HA1 3UJ, UK; 5 Department of Radiology, London North West University Healthcare NHS Trust, Northwick Park Hospital, Harrow, HA1 3UJ, UK; 6 Faculty of Medicine, Imperial College London, London, SW7 2BU, UK

## Abstract

Accurate diagnosis of osteomyelitis underlying pressure ulcers is essential, as overdiagnosis exposes patients to unnecessary and prolonged antibiotic therapy, while failure to diagnose prevents successful treatment. Histopathological examination of bone biopsy specimens is the diagnostic gold standard. Bone biopsy can be an invasive procedure, and, for this reason, other diagnostic modalities are commonly used. However, their accuracy is questioned in literature.

This systematic review aims to assess accuracy of various modalities (clinical, microbiological and radiological) for the diagnosis of pelvic osteomyelitis in patients with pressure ulcers as compared to the gold standard.

A systematic literature search was conducted in July 2019 using the MEDLINE (Medical Literature Analysis and Retrieval System – MEDLARS – Online) and CINAHL (Cumulative Index to Nursing and Allied Health Literature) databases. The search terms were “decubitus ulcer”, “pressure ulcer”, “pressure sore”, “bedsore” and “osteomyelitis”. The inclusion criteria were original full-text articles in English comparing the results of bone histology with those of other diagnostic modalities in adult patients with pelvic pressure ulcers.

Six articles were included in the systematic review. Clinical diagnosis was found to be neither specific nor sensitive. Microbiological examination, and in particular cultures of bone biopsy specimens, displayed high sensitivity but low specificity, likely reflecting contamination. Radiological imaging in the form of X-ray and CT (computed tomography) scans displayed high specificity but low sensitivity. MRI (magnetic resonance imaging), bone scanning and indium-labelled scintigraphy displayed high sensitivity but low specificity.

Our systematic review did not find any diagnostic method (clinical, microbiological or radiological) to be reliable in the diagnosis of pelvic osteomyelitis associated with pressure ulcers as compared to bone histology.

## 1 Introduction

Pressure ulcers are caused by injury to the skin and underlying tissue, due to external forces such as pressure and/or shearing forces (Edsberg et al., 2016). They often occur in areas of bony prominence, such as the sacrum (Vanderwee et al., 2007). Patients at risk of pressure ulceration include those with spinal cord injuries (SCIs) and those with limited mobility, such as older people (Gefen, 2014; Bergstrom et al., 1998).

The National Pressure Ulcer Advisory Panel (NPUAP), the European Pressure Ulcer Advisory Panel (EPUAP) and the Pan Pacific Pressure Injury Alliance (PPPIA) have agreed on a definition and categorisation of pressure injuries. These state that pressure ulcers vary in severity from non-blanchable erythema of intact skin (grade 1) and partial-thickness skin loss with exposed dermis (grade 2) to full-thickness skin loss (grade 3) and full-thickness skin and tissue loss (grade 4) (Edsberg et al., 2016).

Pressure ulcers carry substantial morbidity and present a significant financial burden for healthcare systems. Between April 2015 and March 2016, 24 674 patients were reported to have developed a new pressure ulcer in the NHS (National Health Service) in England (NHS Improvement, 2018). In 2012, the estimated cost of treating a pressure ulcer varied between GBP 1214 (grade 1) and GBP 14 108 (grade 4) (Dealey et al., 2012), while the estimated NHS expenditure for treating pressure damage amounts to more than GBP 3.8 million per day (NHS Improvement, 2018).

Osteomyelitis can develop in bone underlying pressure ulcers. Treatment of osteomyelitis is complex and often involves prolonged antibiotic courses of 6 or more weeks and repeated surgical procedures. In order to optimise outcomes and treat possible recurrence, these patients need to be treated in a specialised institution by a dedicated interdisciplinary team including infectious-disease clinicians, orthopaedic and plastic surgeons (Dudareva et al., 2017).

Accurate diagnosis of osteomyelitis underlying pressure ulcers is essential, as overdiagnosis exposes patients to unnecessary and prolonged treatment with antibiotics, while failure to diagnose prevents successful treatment. Histopathological examination of bone biopsy specimens is the gold standard for diagnosis. However, bone biopsy can be an invasive procedure and requires the involvement of surgeons in order to be carried out optimally with the collection of multiple specimens for microbiological and histopathological examination.

For this reason, alternative diagnostic modalities are commonly used in clinical practice. These include clinical assessment, microbiological (bone and tissue cultures) and radiological (X-ray, CT – computed tomography – scan, MRI – magnetic resonance imaging, bone scan and scintigraphy) investigations. However, the accuracy of these methods has been questioned in literature (Livesley and Chow, 2002; Wong et al., 2019). This systematic review aims to assess the accuracy of these modalities for the diagnosis of pelvic osteomyelitis in patients with pressure ulcers as compared to the gold standard, i.e. bone histology.

## 2 Methods

For a high standard of reporting, we followed the preferred reporting items for systematic reviews and meta-analyses (PRISMA) guidelines. Details of PRISMA guideline compliance are presented in Appendix A.

### 2.1 Search strategy

The protocol for this systematic review was registered with PROSPERO (International Prospective Register of Systematic Reviews; CRD42019140299). A systematic literature search was conducted in July 2019 using MEDLINE (Medical Literature Analysis and Retrieval System – MEDLARS – Online) and CINAHL (Cumulative Index to Nursing and Allied Health Literature) databases. The search terms used were “decubitus ulcer”, “pressure ulcer”, “pressure sore”, “bedsore” and “osteomyelitis” (see the full search strategy in Appendix B and Appendix C). No date limit was applied.

### 2.2 Selection criteria

Inclusion criteria were as follows: original full-text articles in English comparing the results of bone histology with those of other diagnostic modalities in adult patients with pelvic pressure ulcers. Studies conducted on the paediatric population were excluded, as were studies that did not report bone histology results or comparison with other diagnostic modalities. The search results were screened in order to identify eligible articles; these were read in full and assessed according to the criteria mentioned above. References were also screened, so as to identify further eligible articles. Disagreement was resolved by consensus or, in its absence, by the senior author.

### 2.3 Data extraction

The following data were extracted for each article: author; journal; publication year; study design; number of patients and of pressure ulcers; grade and site; prevalence of osteomyelitis; and, for each diagnostic modality, true positive, true negative, false positive and false negative values, as well as sensitivity and specificity. Both authors extracted data independently.

### 2.4 Outcome measures

The primary outcome was the sensitivity and specificity of the considered diagnostic modalities (clinical, microbiological and radiological) as compared to the gold standard.

**Figure 1:**
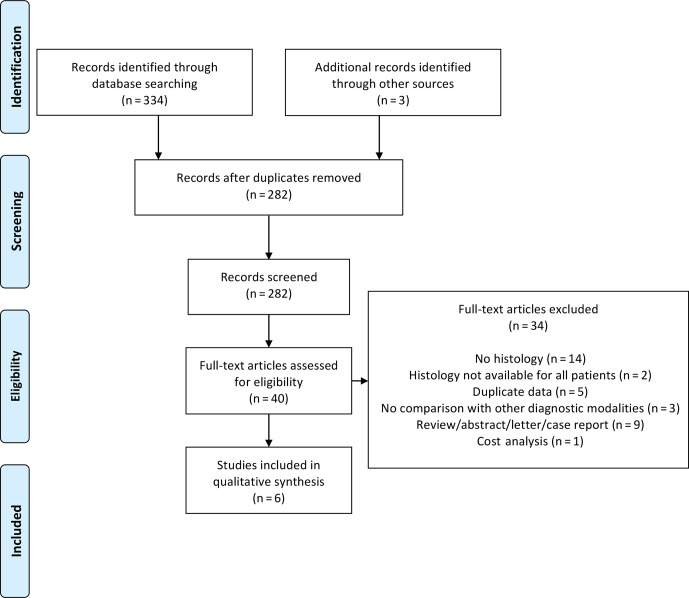
PRISMA diagram.

## 3 Results

### 3.1 Search results

Our systematic search identified 334 articles: 40 articles were read in full, and 6 articles were eventually included (see PRISMA diagram in Fig. 1) – 16 articles were excluded specifically due to the absence of histological data. The six studies included were published between 1986 and 2016 (Thornhill-Joynes et al., 1986; Sugarman, 1987; Lewis et al., 1988; Darouiche et al., 1994; Melkun and Lewis, 2005; Brunel et al., 2016). Most were conducted in the USA and involved retrospective review of patient records (see Table 1).

**Table 1:** Studies included in the systematic review. Columns from left to right: first author, publication year, study design, abbreviated title of the publication journal, country of the study, diagnostic modalities compared in the study and method used to obtain bone biopsy specimens.

Study	Year	Design	Journal	Country	Comparison	Histology
Thornhill-Joynes	1986	Retrospective	Arch Phys Med Rehabil	USA	Histology vs. bone cultures, X-ray, bone scan	Percutaneous bone biopsy withCraig needle or surgical biopsy
Sugarman	1987	Retrospective	Arch Intern Med	USA	Histology vs. clinical examination, bone cultures, X-ray, CT scan, bone scan	Percutaneous bone biopsy withCraig needle
Lewis	1988	Prospective	Plast Reconstr Surg	USA	Histology vs. X-ray, CT scan, bone scan	Percutaneous bone biopsy withJamshidi needle and ostectomy specimen
Darouiche	1994	Prospective	Arch Intern Med	USA	Histology vs. clinical examination, bone cultures, X-ray, bone scan	Percutaneous bone biopsy withCraig needle
Melkun	2005	Retrospective	Ann Plast Surg	USA	Histology vs. tissue cultures, MRI, bone scan, indium-labelled scintigraphy	Percutaneous bone biopsy withJamshidi needle and/or ostectomy specimen
Brunel	2016	Prospective	Clin Microbiol Infect	France	Histology vs. bone cultures Composite criterion (histology and bone cultures) vs. MRI	Surgical bone biopsy

### 3.2 Demographic data

Demographic data are limited, but those available are presented in Table 2: the majority of patients included in the studies were SCI patients, male and comparatively young. One study included only grade 4 pressure ulcers, while two studies included both grade 3 and 4, and three studies did not specifically mention the pressure ulcer grade. Given the small number of studies meeting the inclusion criteria, we decided to retain the six studies irrespective of pressure ulcer grade. Prevalence of osteomyelitis ranged from 17 % to 86 %, with most studies reporting a prevalence of approximately 20 %.

**Table 2:** Patient demographics. n: number. m: mean or median. r: range. SCI: spinal cord injury. CVA: cardiovascular accident.

Study	Patients	Ulcers	Age	Male	Mechanism	Grade	Site	Osteomyelitis
	(n)	(n)	(m,r)	(n, %)				(n, %)
Thornhill-Joynes	40	102	35 (19–65)	35 (87.5)	39 traumatic SCIs 1 paraplegia of unclear aetiology	–	29 ischial 22 sacral 26 trochanteric 25 other	25/102 (24.5)
Sugarman	–	153	–	–	–	32 bone exposed 79 deep tissues exposed 42 more superficial	–	41/153 (27)
Lewis	52	52	–	–	52 SCIs	–	–	12/52 (23)
Darouiche	36	36	–	–	27 SCIs 9 CVAs	15 grade 3 21 grade 4	–	6/36 (17)
Melkun	9	9	49 (22–67)	8 (89)	9 SCIs (6 traumatic)	9 grade 4	3 ischial 2 sacral 4 trochanteric	2/9 (22)
Brunel	34	44	51 (45–59)	24 (71)	34 SCIs	5 grade 3 39 grade 4	24 ischial 15 sacral 5 trochanteric	38/44 (86.4)

### 3.3 Histology

Histological diagnosis of osteomyelitis was defined by the presence of inflammatory cells (either polymorphonuclear leucocytes in acute infection or mononuclear leukocytes in chronic infection) – all studies except one (Melkun and Lewis, 2005) specifically mentioned adhering to this definition. Bone biopsies for histological examination were obtained by a variety of methods (see Table 1): one study obtained surgical biopsies, four studies obtained percutaneous biopsies using a Craig or Jamshidi needle and one study obtained either percutaneous or surgical biopsies. Two studies obtained both percutaneous needle biopsies and ostectomy specimens after patients underwent bone excision and soft-tissue reconstruction. Melkun and Lewis noted that “in all cases where both ostectomy specimens and bone biopsy were available, the results were consistent” (Melkun and Lewis, 2005). Lewis et al. (1988) reported a sensitivity of 73 % and specificity of 96 % for needle biopsy as compared to ostectomy.

### 3.4 Clinical examination

Two studies assessed the sensitivity and specificity of clinical examination compared to histology of bone biopsies. In the first study, one infectious-disease clinician assessed patients prior to biopsy: clinical signs suggestive of osteomyelitis included “grossly purulent drainage, advancing erythematous border, or systemic signs of infection attributed to pressure sore” (Sugarman, 1987). The sensitivity and specificity of clinical examination were 22 % and 79 %, respectively. In the second study, one infectious-disease clinician and one orthopaedic surgeon independently assessed patients for ulcer duration, bone exposure, purulent discharge and fever. The sensitivity and specificity of clinical examination were 33 % and 60 %, respectively (Darouiche et al., 1994).

### 3.5 Bone and tissue cultures

Four studies compared bone cultures, defined as bone biopsy specimens used for culture, and histology results: sensitivity ranged from 76 % to 100 %, and specificity ranged from 8 % to 67 % (see Table 3). Brunel et al. (2016) considered a bone culture positive if one sample grew non-commensal bacteria or if three samples grew the same commensal bacteria. Using these criteria, they found good agreement between histology and microbiology (κ=0.55) (Brunel et al., 2016). One study reported on the results of tissue cultures: sensitivity was 100 %, and specificity was 67 % (Melkun and Lewis, 2005).

**Table 3:** Sensitivity and specificity of diagnostic modalities as compared with the gold standard, i.e. bone histology. TP: true positive. TN: true negative. FP: false positive. FN: false negative. Sensitivity = TP/(TP+FN). Specificity = TN/(TN+FP).

Clinical examination	TP	TN	FP	FN	Sensitivity	Specificity	
Sugarman	9	88	24	32	0.22	0.79	
Darouiche	2	18	12	4	0.33	0.6	
Bone cultures	TP	TN	FP	FN	Sensitivity	Specificity	
Thornhill-Joynes	19	1	12	6	0.76	0.08	
Sugarman	40	58	53	0	1	0.52	
Darouiche	6	8	22	0	1	0.27	
Brunel	35	4	2	3	0.92	0.67	
Tissue cultures	TP	TN	FP	FN	Sensitivity	Specificity	
Melkun	2	4	2	0	1	0.67	
X-ray	TP	TN	FP	FN	Sensitivity	Specificity	
Thornhill-Joynes	13	5	5	6	0.68	0.5	
Sugarman	19	40	54	15	0.56	0.43	
Lewis	–	–	–	–	0.18	1	
Darouiche	3	15	15	3	0.5	0.5	
CT scan	TP	TN	FP	FN	Sensitivity	Specificity	
Sugarman	2	5	4	1	0.67	0.56	
Lewis	–	–	–	–	0.11	0.9	
MRI	TP	TN	FP	FN	Sensitivity	Specificity	
Melkun	0	1	3	1	0	0.25	
Brunel	33	2	7	2	0.94	0.22	${}^{*}$ Composite criterion
Bone scan	TP	TN	FP	FN	Sensitivity	Specificity	
Thornhill-Joynes	16	5	5	1	0.94	0.5	${}^{*}$ 6 equivocal excluded
Sugarman	37	30	65	0	1	0.32	
Lewis	–	–	–	–	0.64	0.57	
Darouiche	6	2	28	0	1	0.07	
Melkun	1	0	1	0	1	0	${}^{*}$ 7 indeterminate excluded
Indium-labelled scintigraphy	TP	TN	FP	FN	Sensitivity	Specificity	
Melkun	2	2	2	0	1	0.5	

### 3.6 X-ray and CT

Four studies reported on the sensitivity and specificity of X-ray for the diagnosis of osteomyelitis: sensitivity ranged from 18 % to 68 %, and specificity ranged from 43 % to 100 %. Two studies also performed CT scanning: sensitivity ranged from 11 % to 67 %, and specificity ranged from 56 % to 90 %.

### 3.7 MRI

Two studies assessed the sensitivity and specificity of MRI: sensitivity differed greatly between studies, with one study reporting a value of 94 % and the other reporting 0 %. Specificity on the other hand was similar between studies – 22 % and 25 %. The study reporting a sensitivity of 0 % only included five patients for which both histological and MRI data were available (Melkun and Lewis, 2005). The other study used a composite criterion of histology and bone cultures to diagnose osteomyelitis (Brunel et al., 2016).

### 3.8 Bone scan

Five studies evaluated technetium bone scans as a diagnostic modality. Reported sensitivity ranged from 64 % to 100 %, and specificity ranged from 0 % to 57 %. In two studies, an important number of bone scans were reported as “equivocal” or “indeterminate” and were not considered in the analysis (Thornhill-Joynes et al., 1986; Melkun and Lewis, 2005).

### 3.9 Scintigraphy

One study assessed indium-labelled autologous leukocyte scintigraphy: sensitivity was 100 %, and specificity was 50 %. Three scans were reported as “inconclusive” and excluded from the results (Melkun and Lewis, 2005).

## 4 Discussion

Although osteomyelitis is a recognised serious complication of pressure ulcers, our review shows that little attention is currently focused on the diagnosis of this condition. Of the 40 articles screened in our review, the majority (21 of 40) were published in the 1980s and 1990s. Few studies have been published in the past 2 decades. The paucity of research on this topic has led some authors to describe pressure-ulcer-related osteomyelitis as a “neglected disease of the developed world” (Bodavula et al., 2015).

The gold standard for diagnosis of pressure-ulcer-related osteomyelitis is histological examination of bone biopsy specimens (Livesley and Chow, 2002). Histopathology can distinguish between osteomyelitis – characterised by the presence of inflammatory cell infiltrates – and pressure-related bone changes, such as fibrosis and reactive bone formation, which are inevitably present within grade 4 pressure ulcers even when cortical bone is intact (Türk et al., 2003).

In clinical practice, alternatives to bone histology such as clinical, microbiological and radiological modalities are commonly used. Our review sought to systematically compare results of these alternative modalities with the gold standard in order to evaluate their accuracy and usefulness.

Clinical examination displayed both low sensitivity and low specificity. Osteomyelitis can be difficult to distinguish clinically from the infection of soft tissues; at the same time, soft-tissue involvement may underestimate the degree of underlying bone involvement, as the pressure exerted on the skin is distributed over a wider bone surface (Livesley and Chow, 2002).

Pressure-ulcer-related osteomyelitis is often polymicrobial and can be caused by gram negative and anaerobes, as well as more usual bacteria such as *Staphylococcus aureus* and pyogenic streptococci (Sugarman, 1987; Brunel et al., 2016). However, identifying the causative pathogen can be challenging, as pelvic pressure ulcers are often colonised with commensal bacteria of the skin and digestive tract (Deloach et al., 1992). Cultures of bone biopsy specimens represent the cornerstone of osteomyelitis treatment: isolation of the same bacteria from multiple intra-operative samples is essential to target antibiotic therapy.

For diagnostic purposes, cultures of bone biopsy specimens in our review displayed high sensitivity but low specificity, likely reflecting contamination. It is important to note, however, that only one of the included studies reported a strict antibiotic-free period before bone biopsy (Brunel et al., 2016). Bone sampling methods and microbiological interpretation criteria of cultures to distinguish between causative bacteria and contamination varied across studies. These factors may have affected the accuracy of bone cultures as a diagnostic modality in our study. Using multiple specimens with stringent microbiological criteria for the collection, processing of specimens and interpretation of culture results, Brunel et al. (2016) found good agreement between bone cultures and histology. The role of improved microbiological culture methods, such as sonification to dislodge biofilms and non-culture methods to detect 16S ribosomal RNA (which are used for diagnosis of prosthetic-joint infection), have not been studied in the context of pelvic osteomyelitis secondary to pressure ulcers.

Radiological imaging in the form of X-ray and CT scans displayed high specificity but low sensitivity. False negative rates can be high, in particular with X-ray, as early bone erosion may not be detected (Thornhill-Joynes et al., 1986). On the other hand, MRI, bone scanning and indium-labelled scintigraphy displayed high sensitivity but low specificity. In fact, these imaging techniques may not be able to distinguish between osteomyelitis, soft-tissue inflammation and pressure-related bone changes, e.g. cortical bone erosion and bone marrow oedema (Ruan et al., 1998; Wheat, 1985).

Our systematic review suggests that no diagnostic modality offers a sufficiently accurate alternative to bone histology, which remains the gold standard. Therefore, diagnosing pressure-ulcer-related osteomyelitis on the basis of any modality other than bone histology runs the risk of over- or underdiagnosis. While overdiagnosis can expose patients to unnecessary and prolonged antibiotic treatment, failure to diagnose osteomyelitis can jeopardise the successful treatment and healing of pressure ulcers (Han et al., 2002).

A recent study showed that diagnostic approaches to pressure-ulcer-related osteomyelitis vary significantly among infectious-disease clinicians, an important proportion of whom reported low confidence in making this diagnosis. The authors suggest that this reflects the lack of evidence and of agreed diagnostic criteria for this condition (Kaka et al., 2019). This is in contrast with osteomyelitis associated with diabetic foot ulcers, where recognised diagnostic criteria exist and recommendations include performing transcutaneous or surgical bone biopsy for histological and microbiological examination (Lipsky et al., 2012).

Our review has several limitations: a small number of studies, mostly of retrospective design, including a low number of patients. The majority of studies were conducted in SCI patients, and this may affect the generalisability of results to older people, who represent the main population affected by pressure ulcers. Furthermore, while it is generally agreed that osteomyelitis develops in grade 4 pressure ulcers, the selected studies included both grade 3 and 4 pressure ulcers or did not specify pressure ulcer grade; this may further affect the generalisability of our results.

## 5 Conclusions

In our systematic review, we did not find any alternative diagnostic method (clinical, microbiological or radiological) to be reliable in the diagnosis of osteomyelitis associated with pelvic pressure ulcers.

Clinical diagnosis is neither specific nor sensitive. Microbiological examination, in particular, bone cultures, displayed high sensitivity but low specificity, likely reflecting contamination. Use of multiple bone specimens collected appropriately, processed using agreed protocols with culture results interpreted using validated criteria, may increase the accuracy of microbiological examination. Radiological imaging in the form of X-ray and CT scans displayed high specificity but low sensitivity. MRI, bone scanning and indium-labelled scintigraphy displayed high sensitivity but low specificity.

To avoid unnecessary and prolonged antibiotic therapy or a failure to treat osteomyelitis, it is important that clinicians should be aware of the limitations of clinical and radiological diagnostic modalities. Microbiological examination is also unreliable unless it is undertaken appropriately using bone biopsies. Further research is necessary to identify improved strategies for the accurate diagnosis of osteomyelitis in patients with pelvic pressure ulcers.

## Data Availability

All data are presented in the tables and figures.

## Appendix A

**Table A1:** PRISMA checklist.

Section or topic	No.	Checklist	Reported on
		item	page no.
TITLE			
Title	1	Identify the report as a systematic review, meta-analysis or both.	1
ABSTRACT			
Structured summary	2	Provide a structured summary including, as applicable, background; objectives; data sources; study eligibility criteria, participants and interventions; study appraisal and synthesis methods; results; limitations; conclusions and implications of key findings; and systematic-review registration number.	1
INTRODUCTION			
Rationale	3	Describe the rationale for the review in the context of what is already known.	2
Objectives	4	Provide an explicit statement of questions being addressed with reference to participants, interventions, comparisons, outcomes and study design (PICOS).	2
METHODS			
Protocol and registration	5	Indicate if a review protocol exists and if and where it can be accessed (e.g. web address), and, if available, provide registration information including the registration number.	3
Eligibility criteria	6	Specify study characteristics (e.g. PICOS and length of follow-up) and report characteristics (e.g. years considered, language and publication status) used as criteria for eligibility, giving rationale.	3
Information sources	7	Describe all information sources (e.g. databases with dates of coverage and contact with study authors to identify additional studies) in the search and date last searched.	3
Search	8	Present full electronic search strategy for at least one database, including any limits used, such that it could be repeated.	Appendix B,Appendix C
Study selection	9	State the process for selecting studies (i.e. screening, eligibility, if included in systematic review and, if applicable, included in the meta-analysis).	3
Data collection process	10	Describe the method of data extraction from reports (e.g. piloted forms, independently, in duplicate) and any processes for obtaining and confirming data from investigators.	3
Data items	11	List and define all variables for which data were sought (e.g. PICOS and funding sources) and any assumptions and simplifications made.	3
Risk of bias in individual studies	12	Describe the methods used for assessing risk of bias of individual studies (including specification of whether this was done at the study or outcome level) and how this information is to be used in any data synthesis.	3
Summary measures	13	State the principal summary measures (e.g. risk ratio and difference in means).	3
Synthesis of results	14	Describe the methods of handling data and combining results of studies, if done, including measures of consistency (e.g. I${}^{2})$ for each meta-analysis.	Table 3
Risk of bias across studies	15	Specify any assessment of risk of bias that may affect the cumulative evidence (e.g. publication bias and selective reporting within studies).	4–5
Additional analyses	16	Describe methods of additional analyses (e.g. sensitivity or subgroup analyses and meta-regression), if done, indicating which were pre-specified.	n/a
RESULTS			
Study selection	17	Give numbers of studies screened, assessed for eligibility, and included in the review, with reasons for exclusions at each stage, ideally with a flow diagram.	3
Study characteristics	18	For each study, present characteristics for which data were extracted (e.g. study size, PICOS and follow-up period), and provide the citations.	Table 1
Risk of bias within studies	19	Present data on risk of bias of each study and, if available, any outcome level assessment (see item 12).	4–5
Results of individual studies	20	For all outcomes considered (benefits or harms), present, for each study, (a) simple summary data for each intervention group and (b) effect estimates and confidence intervals, ideally with a forest plot.	4–5
Synthesis of results	21	Present results of each meta-analysis done, including confidence intervals and measures of consistency.	n/a
Risk of bias across studies	22	Present results of any assessment of risk of bias across studies (see item 15).	4–5
Additional analysis	23	Give results of additional analyses, if done (e.g. sensitivity or subgroup analyses and meta-regression; see item 16).	n/a
DISCUSSION			
Summary of evidence	24	Summarise the main findings including the strength of evidence for each main outcome; consider their relevance to key groups (e.g. healthcare providers, users and policymakers).	5–6
Limitations	25	Discuss limitations at the study and outcome level (e.g. risk of bias) and at the review level (e.g. incomplete retrieval of identified research and reporting bias).	6
Conclusions	26	Provide a general interpretation of the results in the context of other evidence and implications for future research.	6–7
FUNDING			
Funding	27	Describe sources of funding for the systematic review and other support (e.g. supply of data) and the role of funders for the systematic review.	1

n/a – not applicable

## Appendix B

**Table B1:** Search strategy and results from CINAHL until 1 July 2019. Exp: exploded. ADJ: adjacency. ti,ab: terms in the title or abstract fields.

Steps	Search term	Items found
No. 1	exp “PRESSURE ULCER”/	12 361
No. 2	(bed ADJ sore*).ti,ab	73
No. 3	(bedsore*).ti,ab	161
No. 4	(decubitus ADJ ulcer*).ti,ab	397
No. 5	(pressure ADJ ulcer*).ti,ab	7489
No. 6	(pressure ADJ sore*).ti,ab	1569
No. 7	(1 OR 2 OR 3 OR 4 OR 5 OR 6)	14 084
No. 8	OSTEOMYELITIS/	3143
No. 9	(osteomyelitis).ti,ab	3370
No. 10	(8 OR 9)	4464
No. 11	(7 AND 10)	106
No. 12	11 [DT 1970–2019] [Languages eng]	104

## Appendix C

**Table C1:** Search strategy and results from MEDLINE until 1 July 2019. ADJ: adjacency. ti,ab: terms in the title or abstract fields.

Steps	Search term	Items found
No. 1	“PRESSURE ULCER”/	11 776
No. 2	(bed ADJ sore*).ti,ab	209
No. 3	(bedsore*).ti,ab	475
No. 4	(decubitus ADJ ulcer*).ti,ab	1745
No. 5	(pressure ADJ ulcer*).ti,ab	7041
No. 6	(pressure ADJ sore*).ti,ab	2995
No. 7	(1 OR 2 OR 3 OR 4 OR 5 OR 6)	15 610
No. 8	exp OSTEOMYELITIS/	22 128
No. 9	(osteomyelitis).ti,ab	21 122
No. 10	(8 OR 9)	29 394
No. 11	(7 AND 10)	273
No. 12	11 [DT 1970–2019] [Languages English]	230
